# Medicinal ethnoveterinary plants used for treating livestock ailments in the omo-gibe and rift valley basins of Ethiopia

**DOI:** 10.1186/s12917-024-04019-6

**Published:** 2024-04-30

**Authors:** Abenezer Wendimu, Elias Bojago, Yitbarek Abrham

**Affiliations:** 1 Biology Department, Wolaita Sodo University, Natural and Computational Sciences College, PO Box 138, Wolaita Sodo, Ethiopia; 2 Environmental Science Department, Wolaita Sodo University, Natural and Computational Sciences College, PO Box 138, Wolaita Sodo, Ethiopia; 3 College of Agriculture, Department of Horticulture, Wolaita Sodo University, PO Box 138, Wolaita Sodo, Ethiopia

**Keywords:** Animal diseases, Ethno-veterinary, Medicinal plants, Traditional medicine

## Abstract

**Background:**

Traditional medical practices have been used to maintain animal health for millennia and have been passed down orally from generation to generation. In Ethiopia also, plants are the primary means by which the indigenous people in remote areas treat the illnesses of their animals. The present study was therefore, carried out to document the type and distribution of medicinal plants of the county.

**Methods:**

To collect ethnobotanical information, a total of 205 informants (133 men and 72 women) were selected. Among these 121 traditional medicine practitioners, while the remaining 84 were selected through a systematic random sampling method. Ethnobotanical data were collected between January 2023 and August 2023 through semi-structured interviews, participant observation, guided filed walks and focus group discussions. Using descriptive statistics, the ethnobotanical data were analyzed for the Informant Consensus Factor (ICF) and Fidelity Level (FL) values, preference, and direct matrix rankings. SPSS 26.0 and PAST 4.11 software were used in data analysis.

**Results:**

Totally, 78 ethnoveterinary medicinal plants distributed in 36 families were identified in the study area. Asteraceae was the dominant family with 9 species (14%), followed by Euphorbiaceae with 8 species (12%). Herbs 42(56%), wild collected 62 (66%), and leaf part (52%) made the highest share of the plant species. *Hordeum vulgare* L. had the highest fidelity level (FL = 98%) for treating bone fractures. Blackleg, bloat, and endoparsistes each had the highest values of the consensus factor among the informants (ICF = 1). According to preference ranking, *Withania somnifera* was the most potent remedy for treating blackleg. Knowledge of medicinal plants was shared through storytelling within families.

**Conclusion:**

In the study area, there is broad access to traditional medicinal plants that can treat ailments in animals. Conservation efforts should be prioritized to protect medicinal plants from threats such as agricultural expansion, drought, and development. Further research should be conducted to explore the potential of different medicinal plants for treating common livestock ailments.

**Supplementary Information:**

The online version contains supplementary material available at 10.1186/s12917-024-04019-6.

## Background

Livestock production is vital to developing nations' rural economies, especially in rural communities [1]. Food security and poverty reduction are two benefits it provides, along with support for many cultural rituals [2]. Animals fulfill a number of social roles and are a major source of food, money, and nutrient-rich dung that may rebuild soil [3, 4]. However, their likelihood of catching other diseases is usually higher. Diseases that affect livestock raising to differing degrees may put different kinds of animals at risk [5–7]. Ethnoveterinary medicine (EVM) is the application of a range of belief-based systems and knowledge, conventional wisdom, experience, techniques, knowledge of medicinal plants, technologies, and teaching to the care of livestock in order to maintain animal health [8]. However, public veterinary services are only available in large cities and some selected area in terms of economic importance [9]. So, for farmers and livestock herders in remote locations, EVM provides a viable replacement for western veterinary procedures. According to McGaw and Eloff et al. [10], plants contain a variety of phytochemicals and investigations in EVM are necessary. Wendimu et al. [11] claimed these plants can be the leading candidates for the creation of medications and other active items that are useful for controlling human and livestock health ailments. As a nation, Ethiopia continues to rely heavily on traditional medicine, and medicinal herbs in particular, to address problems with livestock health [11, 12]. Given that the country is listed in two biodiversity hotspots and has a rapid population growth and the continued loss of biodiversity highlights the necessity of researching plant resources, especially local species that are considered endemic or indigenous [13, 14].

Despite some efforts [12, 15–19], the lack of documenting and dissemination of scientific data on the use of ethnoveterinary medicine among various ethnic groups worldwide represents a crucial gap in the body of material currently available. The majority of diagnostic and treatment expertise has historically been passed down orally and through mentoring; written records have been used sparingly [20–22]. The research and documentation of traditional ethnoveterinary medicine need to be prioritized more and more, as there is a possibility that indigenous knowledge levels could be lost due to many circumstances, including urbanization, migration, and technology [11, 23–27]. The importance of this corpus of information is best expressed by the African saying "When an erudite old man dies, the whole library disappears" [28].

People in the Omo-Gibe and Rift Valley Basins in southern Ethiopia's Wolaita Zone use traditional diagnostic techniques and plant-based treatments to treat livestock ailments. This research investigated ethnoveterinary practices in this region, focusing on traditional diagnostic tools and plant-based treatments for livestock disease, with the goal of preserving indigenous knowledge and improving animal health.

## Materials and methods

### Description of the study area

Wolaita is the name of one of Ethiopia's zonal administrations. The Wolaita people, whose ancestral home is in the zone, gave it their name. Wolaita is bordered by Gamo Gofa on the south, the Omo River on the west, which separates it from Dawro, Kembata, Tembaro on the northwest, Hadiya on the north, the Oromia Region on the northeast, the Bilate River on the east, which separates it from Sidama Region, and Lake Abaya on the south-east, which separates it from Oromia Region. Sodo serves as the administrative hub of Wolaita. Wolaita Zone is located in one of Ethiopia's Southern Regional states. With a surface area of 4383.7 km^2^, the zone has a population above 5.3 million [29].

**Fig. 1:**
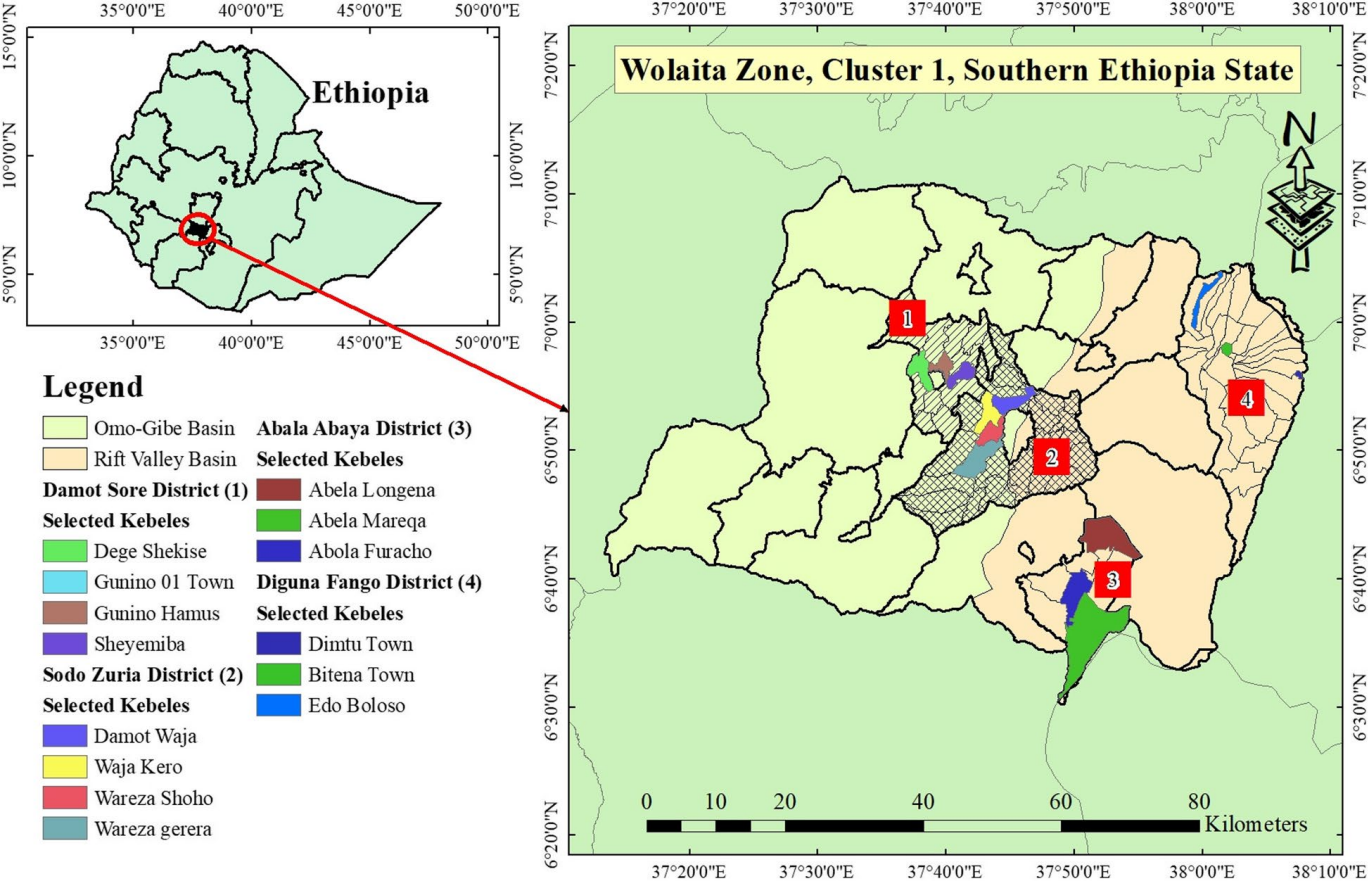
The study area map, Wolaita Zone, Cluster 1, South Ethiopia Regional State (Source: AcrMap10.4.1 by Abenezer Wendimu)

The study was carried out in four districts, such as Damot Sore District and Sodo Zuria District in Omo-Gibe Basin Side and Diguna Fango District and Abala Abaya District in Rift Valley Basin Side of Wolaita Zone (Fig. 1). The maximum monthly temperatures are 17.7°C in July and 22.1°C in February and March. The average annual temperature is 19.9°C [30–32]. The region receives an average of 1,350 mm (53 in) of rainfall annually, according to Bagnara [30].

### Study design

In a cross-sectional study approach was used to collect ethnoveterinary information from traditional healers in the Wolaita zone between January 2023 and August 2023. The primary study parameters were indigenous ethnobotanical knowledge, resources, and their applications.

### Sampling procedure and informant selection

The key informants were chosen by using purposive sampling method. Following the discovery of a few practioners/healers utilizing the sources mentioned above, fruitful initial interactions were made and further ethnopractitioners were found using their existing networks. This method is frequently employed in indigenous knowledge studies to gather data from unexplored communities that are challenging for academics to reach. With the help of the purposive sample technique, it was ensured that only the key respondents with the necessary traits and levels of understanding of the traditional animal healthcare system were selected [32]. Two Hundred Five (205) individuals were chosen to take part in the study, with 65% of them being men and 35% being women while the remaining 84 were selected through a systematic random sampling method. Participants included were farmers, livestock, herders, and individuals with indigenous knowledge, and their ages ranged from 18 to 95. From the total population, 121 were key informants. The selection method for the study's target or key respondents was depending on the target individual's background experience in contemporary ethnoveterinary medical practices. Ethnopractitioners who give the local livestock vital medical care were designated as the target or key respondents.

### Data collection

From Damot Sore District, in Rift Valley Basin side, the kebeles (wards; small administrative unit of the district) used to conduct the research were Dege Shekise, Gunino 01, Gunino Hamus, and Sheyemiba kebeles while in Sodo Zuria District, Damot Waja, Waja Kero, Wareza Shoho, and Wareza Gerera kebeles. In Rift Valley Basin side, Abela Longena, Abela Mareqa and Abela Gurcho were kebeles selected from Abala Abaya District and Dimtu Town, Bitena Town and Edo Boloso were kebeles selected from Diguna Fango District.

An open interview questions was created for data collection and given to the participants. The interview was split into two segments. Basic data, including the respondent's name, age, gender, place of birth, and level of education, were gathered in the first section. Different items made up the open interview in the second section including the names of plants, a list of ailments/diseases treated with the claimed MPs, plant parts utilized, mode of preparation, plant habitat, source of information acquisition, etc. The interviewees were requested to go with us to the neighboring mountains, farms, or grasslands to identify the medicinal plants they used and to offer local colloquial names in Wolaita dialect, in addition to written records and audio recordings, which were taken with another party's permission. Individual interview data were cross-checked with additional participants' commentaries from the same villages in order to gather trustworthy information about the study region [33].

### Questionnaire distribution

The interviewer assisted each of the respondents in completing a well-structured questionnaire (S1 Appendix). The survey consisted of 18 questions covering: village, respondent's information, approval, ethnoveterinary medicine details, animal species treated, remedy identification, care, compensation, knowledge exchange, livestock illnesses treated, plants used, plant state, plant variables, plant actions, challenges, respondent's views, recommendations, and direct observation by the interviewer.

The interviewer was required to be accompanied by a senior relative or friend, as well as a member of the local government from the office of the area subchief who was acquainted with the interviewee, whenever a questionnaire was delivered to the subject. As the interviewer completed the questionnaire, these two individuals actively and productively engaged the interviewee in conversation. This combination produced a highly beneficial interaction that created a favorable setting for the successful completion of research using Rapid Rural Appraisal (RRA) and Participatory Rural Appraisal (PRA). Because it lessened the following causes of bias, this technique was deemed to be very effective and reliable:—(1) modeling bias, or projecting the interviewer's viewpoint onto the subjects under study, (2) strategic bias, or the subject's expectation of benefits, (3) relationships between senior relatives, administrator representatives, and interviewees that are familiar with one another may lead to rote responses and outsider bias while lessening resistance to questioning and (4), decreasing the influence of "key personas" [25]. Due to these preconceived notions, questionnaires would be filled out incorrectly and the data collected would not be properly documented or analyzed [12, 15].

### Plant specimen collection and identification

After the key respondents were personally interviewed, the indicated specimens of plants were identified and collected during numerous field trips. The specimens were collected, and each plant species was verified using The World Flora Online [34] and The Plant List [35] before botanical identification was completed in a field laboratory using biological keys. They were photographed and sampled for identification at the National Herbarium of Ethiopia. Formal identification of the plant materials were carried out by Abenezer Wendimu (the first author) and Zekarias Demisseie (a botanist) assisted the identification.

### Data analysis

The collected data on medicinal plants, their uses, and related knowledge were recorded and coded on Microsoft Excel. Descriptive statistics like percentages and frequency tables were employed to analyze the data using SPSS version 20.Remarks about the medical worth of plants, their growing forms, modes of preparation, how they are used, routes of adminstration, dose, and what components of the plants they are made of were all part of the interview session. Using the t-test at a 95% confidence level, it was enabled to us to compare the traditional medical dynamics about the use of plants for medicinal purposes by men and women, young and old, illiterate and educated, key and general informants.

According to Chekole et al. [36], quantitative ethnobotanical methods like Informant Consus Factor (ICF) values were calculated to determine the most common livestock illnesses categories reported across the communities and to identify potentially effective medicinal plant species in respective disease categories.

$${\text{ICF}}=\frac{{{\text{N}}}_{{\text{ur}}}-{{\text{N}}}_{{\text{t}}}}{{{\text{N}}}_{{\text{ur}}}-1}$$

where; nur (category's total number of citations) was calculated by subtracting the total number of species utilized (nt) and dividing the result by the total number of citations in each category, minus one.

Furthermore, Fidelity Level (FL) values were computed as follows: FL (%) = Ip/Iu × 100. Where Iu stands for all informants who indicated using a plant to treat any sickness, while Ip indicates how many respondents overall suggested utilizing a plant to treat a particular condition. Preference ranking was developed to determine the effectiveness of particular medicinal plants against the most prevalent diseases in the research district.

## Results

### Diversity of ethnoveterinary medicinal plants in the district

**Fig. 2:**
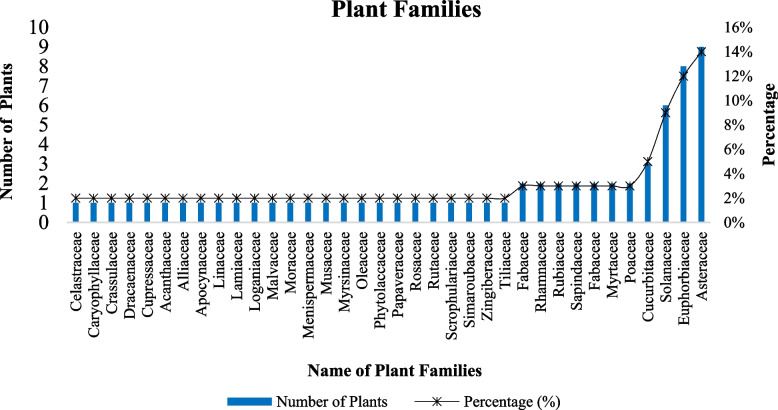
Frequency of the 32 plant families used in the treatment of livestock diseases

The research districts contained 78 different species of ethnoveterinary medicinal plants, which are belongs into 36 families (Table 1). Asteraceae was a dominant family with 9(14%) species followed by Euphorbiaceae 8(12%), Solanaceae 6(9%), and Cucurbitaceae 3(5%) (Fig. 2).

**Table 1:** The diversity of medical plants used in traditional ethno-veterinary practices, the methods used in their production and application to treat typical livestock maladies in Wolaita, South Ethiopia

Family Names	Scientific Name and Voucher Code	English and Local Names (FL)	Growth Form	Collection habits	Used Part	Ailments treated	Remedy Preparation and dosage	Administration Route	Freq	Used by	FL (%)
**Acanthaceae**	*Justicia schimperiana* (Hochst. ex Nees) T., (SW 35)	Justicia (E), Olomuwa (W), Sensel, Simiza, Sansal and Dumoga (A)	Shrub	Wild	Leave	Rabies	Crushing the leaves of the plant and adding in to a hot water. Afterwards, one glass of preparation is given per day for three consecutive days	Oral	By weekly	Bovine, ovine, caprine	17%
						Circling disease	The crushed leaves are mixed with cold water, filtered after 30–40 min and administrated orally	Oral (drinking)	Recovery	Ovine and caprine	54%
**Alliaceae**	*Allium sativum* L., (BN077)	Garlic (E), Nechi shenkurt (A), Shunkurtiya (W)	Herb	Cultivated	Bulb	General illness	When in pain, a combination of crushed *J. procera* leaf and *A. sativum* bulb is administered. For sheep and goats, one liter of the mixture is administered; for cows and oxen, two liters	Oral	Recovery	Bovine	13%
**Aloaceae**	*Aloe spp.* (MNWP-05)	Aloe (E), Wende rate (A), Atuma godarre utta (W)	Herb	Wild	Sap	New castle	The fresh stem of the plant is crushed and squeezed. Then resulting sap is given until recovery	Oral	Recovery	Galine	29%
**Aloaceae**	*Aloe pulcherrima* Gilbert & Sebsebe, (MW-002)	Aloe (E), Sete rate (A), Macca godarre utta (W)	Shrub	Wild	Sap	New castle	The fresh stem of the plant is crushed and squeezed. Then resulting sap is given until recovery	Oral	Recovery	Galine	45%
**Apiaceae**	*Anethum graveolens* L., (MB-53)	Dill (E), Insilaalee (A), Wosoluwa (W)	Herb	Cultivated	Leave	Bloat	Crushed leaf mixed with water and filtered. The, the prepared mixture is given orally to drinking	Oral	Once a day	Bovine	21%
**Apiaceae**	*Diplolophium africanum* Turcz, (GC041)	Yeferse zenge (A)	Herb	Wild	Leave	Taenia	Crushed leaf mixed with water and filtered. The, the prepared mixture is given orally to drinking	Oral	Once	Bovine	19%
**Apocynaceae**	*Carissa spinarum* L., (MB-38)	Agamsa (A)	Shrub		Root	Respiratory allergies	Crushing and adding water	Oral	Once	Bovine, ovine, caprine	17%
**Asteraceae**	*Acmella caulirhiza* Del., (SW 15)	Acmella (E), Aydaamiya (W), Yemdir berbere (A)	Herb	Wild	Leave	Leech	When the plant's fresh leaves are pinched, the fragrance causes gasping and sneezing	Nasal	Once	Bovine	68%
					Seed	Internal parasite	The dry ground seed is mixed with milk in 0.5L bottle	Oral (drinking)	Once	Feline	29%
					Leave	Newcastle	The fresh plant leaves were crushed and soaked with water in open dish for an hours. The sauce is then filtered and only the liquid part is collected in 1L bottle	Oral	Twice	Poultry	31%
**Asteraceae**	*Artemisia afra* Jacq. ex Willd., (SW 17)	African Wormwood (E), Chekugne (A), Cuqqunaiya (W)	Shrub	Wild	Leave	Diarrhea	Crushed fresh plant leaves are blended for five minutes with hot water. After filtration, the produce is then administered orally. The dosage is 1 L each day until the recovery	Oral	Once	Bovine	74%
**Asteraceae**	*Artemisia spp*., (SW 17)	Naatraa (W)	Herb	Cultivated	Stem, Leave	Bloating	When in pain, this plant's fresh shoot or leave and an *E. globulus* leave are crushed together and given in doses of one liter for cows and oxen and half a liter for sheep and goats	Oral	Recovery	Bovine, caprine, ovine	88%
**Asteraceae**	*Conyza spp*.	Horseweed (E), Asfa (A), Chakga (W)	Herb	Wild	Leave	Internal parasite	Crushed plant material and water are combined with the plant's young leaves. Then, one liter of the concoction is administered once daily in the morning	Oral (drinking)	Twice	Bovine	47%
**Asteraceae**	*Echinops* spp., (SW 25)	Glandular globe-thistle (E), Boorisaa (W), Kebericho (A)	Herb	Wild	Root	Diarrhea	The plant's fresh roots are crushed and combined with water. The medication is then administered daily till recovery in quantities of one water glass	Oral (drinking)	Recovery	Bovine	64%
						Pasteurellosis	The plant's freshly formed roots are crushed and combined with water. Then, doses of 1–3 drops of the product are administered	Nasal	Once	Bovine	52%
**Asteraceae**	*Solanecio gigas* (Vatke) C. Jeffrey, (MB-31)	Shecoco gomen (w)	Herb	Wild	Leave	Internal parasite	Crushed fresh plant leaves are blended for five minutes with hot water. After filtration, the produce is then administered orally. The dosage is 1 L each day until the parasite's excretions are removed	Oral (drinking)	Recovery	Bovine	66%
**Asteraceae**	*Spilanthes mauritiana* (A.Rich. ex Pers.) DC. Asteraceae (SW 19)	Spilanthes (E), Aydamiia (W),	Herb	Wild	Leave	Internal parasite	Crushed fresh plant leaves are blended for five minutes with hot water. After filtration, the produce is then administered orally. The dosage is 1 L each day until the parasite's excretions are removed	Oral (drinking)	Recovery	Bovine	66%
**Asteraceae**	*Vernonia amygdalina* Delile, (SW 52)	Bitter leaf (E), Garaa (W), Girawa (A)	Shrub	Wild	Leave	Internal parasite	The young plant leaf is crushed, combined with water, and then consumed. One liter per day until the body returns to its normal state	Oral (drinking)	Recovery	Bovine	47%
**Asteraceae**	Vernonis spp.	Buuzuwa (W)	Shrub	Wild	Flower, Stem Leave	Babesiosis	Crushed flower mixed with water and filtered. The, the prepared mixture is given orally to drinking	Oral (drinking)	Once	Bovine	35%
						Trypanosomiasis	Water is combined with crushed plant leaves. After that, one liter of the prepared juice is administered orally every day till recovery	Oral (drinking)	Recovery	Bovine, ovine, caprine	23%
**Asteraceae**	*Bidens prestinaria* (Sch. Bip.) Cufod, (MB-49)	Chigogot (A)	Herb	Wild	Leave	Insect bite	The fresh plant leaves were crushed and soaked with water in open dish for an hours. The sauce is then filtered and only the liquid part is collected and smeared	Dermal	Once	Bovine, caprine	15%
**Asteraceae**	*Inula confertiflora* A. Rich, (NA89)	Woyina gift (A)	Shrub	Wild	Leave	Eye infection	The dry leaves are ground, powdered, and smeared on the eye	Ocular	Once	Bovine	66%
						Wound	The fresh leaves are crushed, squeezed, and the juice is dropped on the victim area	Dermal	Once	Bovine, caprine	57%
**Brassicaceae**	*Brassica nigra* (L.) K.Koch, (BN089)	Oats (E), Senafich (A),Santta ayfiya (W)	Herb	Wild/cultivated	Seed	Bloat	The plant's dried seeds are crushed and combined with water. Until recovery, a dose of one glass of water per day is administered	Oral	Recovery	Bovine, ovine, caprine	71%
**Brassicaceae**	*Lepidium sativum* L. (SW 34)	Garden cress (E), Sibbikka (W), Fetto (A)	Herb	Wild/cultivated	Seed	Bloat	By grinding the dried seeds of the plant, the powder is mixed with water, it is good if the water is hot but ok with cold also. One water glass of the preparation is given every day for a week	Oral	Once everyday	Bovine, ovine, caprine, bovine	23%
**Caryophyllaceae**	*Silene macrosolen* A. Rich	Silene (E), Wegert (A)	Herb	Wild	Whole body	General illness	Water and the ground-up plant root are combined. The combination is then heated for a short period of time on a stove. After the juice has cooled, it is subsequently introduced three to five drops into each nostril	Nasal	Twice a week	Bovine	33%
**Celastraceae**	*Maytenus senegalensis* (Lam.) Exell	Qoqoba (A)	Shrub	Wild/cultivated	Leave	Insect bite	The fresh plant leaves were crushed and soaked with water in open dish for an hours. The sauce is then filtered and only the liquid part is collected and smeared	Dermal	Once	Bovine, ovine, caprine	52%
**Crassulaceae**	Kalanchoe spp,	Flaming Katy (E), Mul’uwa (W)	Herb	Wild	Root, Leave	Abscess	The fresh root cover is collected from plant and applied on the swellings	Topical	Twice	Bovine	38%
						Erectile dysfunction ED	The plant's young leaves are crushed and combined with hot water. Following that, the frigid solution is given orally	Oral (drinking)	Once	Bovine	14%
**Cucurbitaceae**	*Cucumis ficifolius* A. Rich, (MB-15)	Cucumbers (E), Ymider enboye (A)	Herb	Wild	Root	Blackleg	Crushed plant roots are combined with cold water. Then, one glass of the solution is administered orally every day until healing has occurred	Oral	Recovery	Bovine	29%
**Cucurbitaceae**	*Lagenaria siceraria* (molina) standl. (SW 55)	Bottle gourd (E), Gosiya (W), Kil (A)	Herb	Wild	Fruit	Rabies	Every day for a week, animals are given a half-liter of a mixture made from the crushed fruit of this plant and the leaves of *P. dodecandra*, together with salt and water	Oral	Recovery	Bovine, ovine, caprine	12%
**Cucurbitaceae**	*Mukia maderaspatana* (L.) M.J. Roem	Yeamora misa (A)	Climber	Wild	Leave	Mite infestation	Soaking the fresh leaves of the plant in water. Thereafter, washing the victim site with one water glass of solution per day until parasite load lessens	Dermal	Recovery	Bovine	25%
**Cupressaceae**	*Juniperus procera* Hochst. Ex Endl. (SW 56)	African juniper (E), Abeshaa xidaa (W), Tsid (A)	Tree	Wild	Leave	Ectoparasites infestation	Soaking the fresh leaves of the plant in water. Thereafter, washing the victim site with one water glass of solution per day until parasite load lessens	Topical	Recovery	Bovine, ovine, caprine	67%
						Bloating	When in pain, a combination of crushed *J. procera* leaf and *A. sativum* bulb is administered. For sheep and goats, one liter of the mixture is administered; for cows and oxen, two liters	Oral	Once	Bovine, ovine, caprine	32%
**Dracaenaceae**	*Sansevieria ehrenbergii* Schweinf. Ex Baker, (GC111)	Snake Plants (E), Wende kacha (A)	Shrub	Wild	Leave	Mite infestation	Soaking the fresh leaves of the plant in water. Thereafter, washing the victim site with one water glass of solution per day until parasite load lessens	Dermal	Recovery	Bovine	77%
**Euphorbiaceae**	*Acalypha* spp.	Love-lies-bleeding (E), Gagabissa (W), Lalisho (A)	Herb	Cultivated	Root	Bloat	The fresh or dried root is ground and soaked with water in 1L bottle	Oral	Twice	Bovine	92%
**Euphorbiaceae**	*Croton macrostachyus* Del. (SW 05)	Woodland croton (E), Anka (W), Bisana (A)	Tree	Wild	Leave	Trauma	Until healing, a tiny amount of powder made from the plant's dried and roasted leaf is applied daily to the victim locations	Dermal	Recovery	Goat, sheep and livestock	34%
						Foot rot	The fresh leaves of the plant is crushed and mixed with water. The preparation is then given in doses of one water glass every day in evening for seven consecutive days until recovery	Oral and topical	7 days	Bovine, ovine, caprine	35%
						Blackleg	Crushed fresh leaves of the plant is mixed with water and filtered. The preparation is then given in doses of one liter once a day	Oral (drinking)	Once	Bovine	22%
**Euphorbiaceae**	*Euphorbia abyssinica* J.F.Gmel. (AK 073)	Candelabra Spurge (E), Akkirssaa (W), Qulqual (A)	Herb	Wild	Latex	Rabies	Fresh *Euphorbia abyssinica* latex is pulverized and either administered topically or ingested	Oral	Once	Bovine, ovine, caprine	44%
**Euphorbiaceae**	*Euphorbia tirucalli* L. (SW 57)	Indian tree spurge (E), Maaxxuwa (W), Kinchib (A)	Herb	Wild	Stem	Strangle burning	The fresh stem of the plant is crushed and boiled in hot water. Animals are fumigated with the resulting steam vapor per day until recovery	Fumigation	Once	Equine	50%
**Euphorbiaceae**	*Manihot esculenta* Crantz. (SW 10)	Cassava (E), Mitta boyyiya (W), Kassba (A)	Shrub	Cultivated	Leave	Cough	*Manihot esculenta* fresh leaves are squeezed with water, and the juice is specifically administered orally for chicken	Oral	Once	Galine	37%
**Euphorbiaceae**	*Ricinus communis* L. (SW 47)	Castor oil plant (E), Qobuwa (W), Gulo (A)	Herb	Wild/cultivated	Leave	Foot and mouth disease	Crushed fresh plant leaves are combined with water. Up until the animal is healed, one cup of the prepared solution is administered orally each day	Oral	Once everyday	Bovine	43%
**Euphorbiaceae**	Tragisa spp.	Tinttilashuwa (W)	Herb	Wild	Root Leave	Blackleg	The pulverized dry plant root or fresh plant leaf is combined with water, and the resulting preparation is then administered orally one liter every day till recovery	Oral (drinking)	Recovery	Bovine	14%
**Fabaceae**	*Calpurnia aurea* (A it.) Benth., (MB-39)	Cape laburnum (E), Mello (W), Digita (A)	Shrub	Wild	Leave	Trypanosomiasis	The plant's fresh leaves are crushed and combined with water. Then, 3 to 4 drops of the preparation are administered	Nasal (drop), Oral (drinking)	Every day until recovery	Ovine, bovine	49%
						Mite infestation	The plant's fresh leaves are crushed and the sap is anointed on the infested areas	Dermal		Bovine	65%
**Fabaceae**	*Lonchocarpus laxiflorus* Guill. & Perr	Amera (A)	Tree	Wild	Root, leaf	Listeriosis	Crushed root/leaf mixed with water. One glass of the prepared slimy product is administrated orally till recovery	Oral	Recovery	Caprine	28%
**Fabaceae**	*Millettia ferruginea* (Hochst.) Bak	Pongamia (E), Zagiya (W), Birbira (A)	Tree	Wild	Root	Trypanosomiasis	Crushed root mixed with water. One glass of the prepared slimy product is administrated orally till recovery	Oral (drinking)	Recovery	Bovine	20%
**Fabaceae**	*Trigonella foenum-graecum* L., (BN079)	Fenugreek (E), Abishii (A), Abishiiya (W)	Herb	Wild/cultivated	Seed	Endoparsistes infestation	The dried plant seed is ground the powder is soaked in water. One glass of the prepared product is prescribed orally per day until body condition improved	Oral	Recovery	Bovine, ovine, caprine	77%
**Lamiaceae**	*Ocimum lamiifolium* Hochst. Ex Benth., (SW 30)	Ocimum (E), Gulluwa (W), Damakessie (A)	Shrub	Wild/cultivated	Leave	Febrile illness	The plant's young leaves are crushed and boiled in hot water. Until recovery, one glass of the preparation is administered	Oral	Recovery	Bovine, ovine, caprine	14%
**Linaceae**	*Linum usitatissimum* L., (SW 44)	Linseed (E), Talbbaa (W), Telba (A)	Herb	Cultivated	Seed	Retained placenta	The plant's dried seeds are crushed and boiled for 5–7 min in hot water. Until recovery, one glass of the prepared slimy product is administered orally	Oral	Recovery	Bovine, ovine, caprine	39%
**Loganiaceae**	*Buddleja polystachya* Fresen	Buddlea (E), Kanbara (W), Anfar (Atquar) (A)	Shrub	Cultivated	Leave	Internal parasite	The plant's fresh leaves are crushed and combined with water. The medication is subsequently administered once daily in the morning in quantities of one liter	Oral (drinking)	Recovery	Bovine	47%
**Malvaceae**	*Sida schimperiana* Hochst. ex A. Rich	Sida (E), Kinddiichchuwa (W), Garida (A)	Herb/Shrub	Wild	Leave	Blackleg	Water is combined with freshly crushed plant leaves. Following that, two to five drops of the prepared juice are then ingested through the nose each day until recovered	Nasal (drop)	Recovery	Bovine	67%
**Menispermaceae**	*Stephania abyssinica* (Dillon & A. Rich.) Walp., (MB-13)	Kalala Vine (E)	Herb	Wild	Root	Mastitis	Water and the ground-up plant root are combined. The combination is then heated for a short period of time on a stove. After the juice has cooled, it is subsequently introduced three to five drops into each nostril	Oral (drinking) or Nasal (drop)	Once everyday	Bovine	64%
**Moraceae**	*Ficus vasta* Forssk, (GC162)	Warka (A), Wolla (W)	Tree	Wild	Stem bark	Thrips	The plant's fresh stems is pounded and boiled, thereafter the medication is administered right away	Oral	Once	Bovine, caprine	19%
**Musaceae**	*Ensete ventricosum* (Welw.) Cheesman*,* (SW 07)	Ethiopian banana (E), Utaa (W), Enset (A)	Herb	Cultivated	Whole plant	Eye infection	The whole plant or a fresh leaf is cut or crushed into thin slices. Then, until recuperation, the preparation is provided to eat each day	Ophthalmic	Once/Twice	Bovine, ovine, caprine	78%
					Leave	Retained placenta	Fresh leaves from this plant are fed to animals immediately in order to help them expel the placenta	Oral	Once	Bovine, ovine	55%
					Stem	Retained placenta	Alternatively, a *C. arabica* leaf and its stem are crushed, cooked, and given to sheep and goats in doses of 2–3 L each	Oral	Once	Bovine, ovine, caprine	67%
**Myrsinaceae**	*Maesa lanceolata* Forssk, (GC068)	Gegechuwa (W), Kilabo (A)	Shrub	Wild	Leave	Bloat	Crushed leaves mixed with water. One glass of the prepared slimy product is administrated orally till recovery	Oral (drinking)	Recovery	Bovine	74%
**Myrtaceae**	*Eucalyptus camaldulensis* Dehnh, (AA115)	The river red gum (E), Keye baherzafe (A), Habasha zaafiya (W)	Tree	Wild	Leave	General illness	Fresh leaves are crushed, squeezed, and the juice is given nasally	Nasal	Recovery	Bovine	18%
**Myrtaceae**	*Eucalyptus globulus* Labill. (SW 06)	Tasmanian blue gum (E), Paranjjaa zaafiya (W), Nech baherzaf (A)	Tree	Wild	Leave	Febrile illness	The plant's young leaves are ground up and boiled in hot water. The generated steam vapor is used to fumigate animals once day until they are healthy	Fumigation	Once	Bovine, ovine, caprine	66%
**Oleaceae**	*Jasminum abyssinicum* Hochets. Ex DC., (MA60)	Tero hareg/tenbelele (A)		Wild	Leave	Blackleg	Water is combined with freshly crushed plant leaves. Following that, two to five drops of the prepared juice are then ingested through the nose each day until recovered	Oral	Recovery	Bovine	29%
**Papaveraceae**	*Argemone mexicana* L., (AS-57–2017)	Nechlebash (A)	Herb	Wild	Root	Accident	Water is combined with crushed plant leaves. After that, one liter of the prepared juice is administered orally every day till recovery	Oral	Recovery	Bovine	12%
					Stem	Sore	The fresh plant stem is chopped and sap is anointed on the victim area	Dermal	Recovery	Bovine, ovine, caprine	25%
**Phytolaccaceae**	*Phytolacca dodecandra* L’H´er., (MB-03)	Endod (E), Hancciciaya (W)	Shrub	Wild	Root, Leave	Rabies	Crushed plant roots/leaf are combined with cold water. Then, one glass of the solution is administered orally or nasally every day until healing has occurred	Oral and nasal	Recovery	Bovine, ovine, caprine	58%
**Poaceae**	*Hordeum vulgare* L., (SW 43)	Barley (E), Banggaa (W), Gebis (A)	Herb	Cultivated	Seed	Bone fractures	Animals are given this plant's boiling or uncooked seed (2–5 kg per day) until they recuperate	Oral	Recovery	Bovine, ovine, caprine	98%
**Poaceae**	*Triticum dicoccon* (Schrank) Schübl., (SW 51)	Emmer (E), Banggaa (W), Sinde (A)	Herb	Cultivated	Seed	Bone fractures	*Triticum dicoccon* dry seeds are served for consumption right away	Oral	Once	Bovine, ovine, caprine	78%
**Polygonaceae**	*Rumex nepalensis* Spreng., (GC029)	Nepal Dock (E), Hotorsa (W), Yewsha Tult (A)	Herb	Wild	Leave, root	Coughing	The plant's fresh leaves or roots are crushed and combined with water. The juice mixture is then dissolved in a cup of water and administered orally during coughing seasons	Oral (drinking)	Recovery	Bovine	68%
**Polygonaceae**	*Rumex nervosus* Vahl, (MB-25)	Curly dock (E), Anchechiya (W), Embuachew (A)	Herb	Wild	Stem	Constipation	Twice a week, new fiber from the plant's crushed stem is combined with water and administered orally. The dosage varies depending on the age of the livestock being treated, but in most cases, a half-liter water bottle is the ideal amount	Oral (drinking)	Recovery	Bovine	74%
					Seed	Swelling	The victim region is treated with a liquid that has been made by combining a crushed stem with water and filtering it	Topical application to the wound after opening the swelling	Recovery	Bovine, ovine, caprine	47%
					Leave	External parasites	The dried or fresh plant leave is crushed and the powder or the sauce is applied on the victim site. Has no dosage specificity but the amount and frequency of application depends on the area of infested. High infestation has high application rate and frequencies and vice versa	Topical application	Recovery	Bovine	26%
**Ranculucaceae**	*Ranculucus multifidusfors*.sk	Buttercup (E), Selo (W)	Herb	Wild	Leave	Trypanosomiasis	Crushed fresh plant leaves are combined with water. Next, the prepared remedy is administered orally. In order for the animal to recuperate, the prescribed dosage must surpass 3 to 4 cups of coffee each day	Oral (drinking)	Once	Bovine	15%
**Ranunculaceae**	*Nigella sativa* L., (BN078)	Black cumin (E), Karetta sawuwa/shuquwaa (W), Tikur azmud (A)	Herb	Cultivated	Seed	Colic	The plant's seed is air dried before being pulverized. The powder is then combined with water. Until the symptom goes away, one glass of the prepared product is administered orally	Oral	Once	Bovine, ovine, caprine	55%
**Rhamnaceae**	*Helinus mystacinus* (A it.) E. Mey. ex Steud., (MM097)	Helinus (E), Gofe Gofa (W), Yegib mirkuz (A)	Shrub	Wild	Root	Blackleg	Every day until recovery, the plant's fresh roots are crushed and given to be eaten. Animal are not forced to eat but as per their eating capacity	Oral (drinking)	Recovery	Bovine	25%
**Rhamnaceae**	*Rhamnus prinoides* L’H´er., (SW 46)	Glossy-leaf (E), Geeshuwa (W), Gesho (A)	Shrub	Wild/cultivated	Leave	Rabies	Crushed fresh plant leaves are combined with water. Until the animal feels better, one glass of the prepared solution is administered orally once per day	Oral	Once a day	Bovine, ovine, caprine	14%
**Rosaceae**	*Prunus africana* (Hook. f) Kalkm, (AW-009)	African cherry (E), Onta (W)	Tree	Wild	Leave	Blackleg	Crushed plant leaves are combined with cold water. Following filtration, 1–3 drops of the preparation are administered nasally	Nasal (drop)	Everyday till recovery	Bovine	74%
**Rubiaceae**	*Coffea arabica* L., (SW 03)	Arabian coffee (E), Tukkiya (W), Buna (A)	Shrub	Wild/cultivated	Seed	Wound	Until healing, a tiny amount of powder made from the plant's dried and roasted seeds is applied daily to the victim locations	Topical application	Recovery	Bovine, caprine, ovine	67%
						Retained placenta	To expel the placenta, cows are given 2–3 L of crushed, boiled *E. ventricosum* stem and *C. arabica* leaf mixture, and sheep and goats are given one liter	Oral (drinking)	Recovery	Bovine, ovine, caprine	44%
**Rubiaceae**	*Pentas shemperina* Hiern, (EM-120)	Pentas (E), Dambburssaa (W), Maraxalliya (W), Yejib mirkuz (A)	Herb	Wild	Leave, root, stem	Blackleg	Filtered water was combined with crushed leaves and stem. Until recovery, one glass of the prepared slimy product is administered orally	Oral (drinking)	Once	Bovine	23%
					Leave	Bone fractures	The plant's young leaves are crushed and squeezed. Then hot water is used to boil the liquid. Depending on the severity of the condition, the frigid, thick liquid is then filtered and administered orally once or twice a day	Oral	Once or twice a day	Bovine	56%
**Rutaceae**	*Ruta chalepensis* L., (SW 33)	Fringed rue (E), Xalotiya (W), Tenadam (A)	Shrub	Cultivated	Leave/Seed	Febrile illness	Water is combined with freshly crushed plant leaves or dried seed powder. For the duration of the animal's recovery, one cup of the prepared solution is administered orally every day	Oral and nasal	Once everyday	Bovine, ovine, caprine	33%
**Sapindaceae**	*Dodonaea angustifolia* L.f., (MB-47)	Kitkita (A), Sankarra (W)	Shrub	Wild	Leave	Bone dislocation	Crushed plant stems or tubers are immersed in salted water after being crushed. The produced juice is then given orally once every day, up to recovery, in a glass or pan	Oral	Recovery	Bovine, ovine, caprine	12%
						Hepatitis	Filtered mixture of water and ground seed mixture is then placed on a hot stove for a little while. The juice is then administered nasally by three to five drops after chilling	Nasal	Once	Bovine	36%
						Rheumatic/ Arthritis	Fresh leaf is crushed, mixed with water, and given orally	Oral	Once	Bovine	41%
**Sapindaceae**	*Cardiospermum corundum* L., (AW-009)	Lesser balloon vine (E), Smeg (A)	Shrub	Wild	Leave	Blackleg	Crushed plant leaves are combined with cold water. Following filtration, 1–2 litter of the preparation are administered orally	Oral	Once	Bovine	71%
**Scrophulariaceae**	*Verbascum sinaiticum* Benth., (SW 29)	The scallop-leaved mullein (E), Yeaheya joro (A)	Herb	Wild	Leave	Myiasis	Fresh leaves are crushed, squeezed, and the juice is dropped on the skin	Dermal	Once	Bovine	81%
					Root	Erection of hair	Filtered water was combined with crushed fresh root. Until recovery, one glass of the prepared liquid product is administered orally	Oral	Once	Bovine, ovine, caprine	62%
					Root, Leave	Abdominal pain	Filtered water was combined with crushed fresh root/leaf. Until recovery, one glass of the prepared liquid product is administered orally	Oral	Once	Bovine, ovine, caprine	29%
**Simaroubaceae**	*Brucea antidysenterica* JF. Mill., (MB-10)	Bitter bark tree (E), Shushale (W), Aballo (Waginos) (A)	Tree	Wild	Leave and seed	Epizootic lymphangitis	The dried ground seed and fresh leave are mixed with water and given in doses of one liter once a day	Let them to eat Oral(drinking)	Twice	Equine	55%
**Solanaceae**	*Capsicum annuum* L., (SW 20)	Bell pepper (E), Qaariya (A), Kariya (A)	Herb	Cultivated	Fruit	Colic	The fresh ripen fruit of the plant is juiced using water as diluent. A dose of one water glass is given	Oral (drinking)	Once	Bovine, caprine, ovine	52%
**Solanaceae**	*Datura stramonium* L., (ET020)	Jimson weed (E), Macharara (W), Laflafuwaa(W), Itse Fars (A)	Herb	Wild	Leave	Systemic illness	The plant's fresh leaves are crushed and put in water for maceration. Every day for two days, the preparation is given	Oral	Twice	Equine	42%
						Skin disease	The pulverized dry leaf is combined with water and applied to the skin	Topical application	Recovery	Bovine, ovine, caprine	18%
**Solanaceae**	*Nicotiana tabacum* L., (SW 45)	Tobacco (E), Tanbuwa (W), Tibahu (A)	Herb	Cultivated	Leave	Leech infestation	Filtered water was combined with crushed leaves. Until recovery, one glass of the prepared liquid product is administered orally. Alternately, the plant leaf may be roasted and the smoke inhaled	Nasal and oral	Once	Bovine, ovine, Caprine	32%
						Trypanosomiasis	Crushed leaves mixed with water and filtered. One glass of the prepared liquid product is administrated orally till recovery. Or, the plant leaf is roasted and the smoke is prescribed nasally	Oral (drinking) Nasal	Once	Bovine	29%
						Coughing	For three days, the leaves of this plant is ground up and combined with water; a single water glass is provided to each cow, ox, donkey, and horse, and half of a water glass to each sheep and goat	Oral	Recovery	Bovine, ovine, caprine	12%
					Leave/Seed	Loss of milk quality	Dairy livestock are given the plant's leaves and seeds, which have been dissolved in water. The prepared food is always fed to dairy cows during milking	Oral	Once	Bovine, ovine, caprine	25%
**Solanaceae**	*Solanum incanum* L., (SW 48)	Thorn apple (E), Buluwa (W), Enboy (A)	Shrub	Wild	Seed	Leech	Filtered mixture of water and ground seed mixture is then placed on a hot stove for a little while. The juice is then administered nasally by three to five drops after chilling	Nasal (drop)	Once	Bovine, caprine	47%
**Solanaceae**	*Withania somnifera* (L.) Dunal, (GC048)	Ashwagandha (E), Gizawa (A)	Shrub	Wild	Leave, Stem	Blackleg	Fresh plant stems or leaves are filtered after being combined with water. The liquid is then administered orally twice till recovery	Oral (drinking), Topical	Recovery	Bovine, caprine	54%
**Solanaceae**	*Lycopersicon esculentum* Mill	Timatim (A), Timatimiya (W)	Herb	Cultivated	Leave	Leech infestation	The plant's fresh stems is pounded and boiled, thereafter until recovery, one glass of the prepared liquid product is administered orally	Oral	Once	Bovine, ovine, caprine	28%
**Tiliaceae**	Triumfetta spp.	Burbark (E), Chyshie (W)	Shrub	Wild	Leave	Trypanosomiasis	The fresh crushed leaves is given to eat orally	Oral (drinking)	Once	Bovine	12%
**Urticaceae**	Girardinia spp.	Nettle (E), Kona (W)	Herb	Wild	Leave, stem, root	Rabies	The plant's fresh leaves, stems, or roots are crushed up and combined with water. After being bitten by a rabid animal, the medication is administered right away	Oral (drinking)	Once	Bovine	36%
					Root	Internal parasite	The plant's new roots are crushed and combined with water. The medication is then administered daily till recovery in quantities of one water glass	Oral (drinking)	Twice	Ovine and caprine	41%
**Urticaceae**	*Urtica simensis* L., (GC179)	Sama (A)	Herb	Wild	Leave	Pasteurellosis	Filtered water was combined with crushed fresh root. Until recovery, one glass of the prepared liquid product is administered orally	Oral	Once	Bovine	71%
**Zingiberaceae**	*Zingiber officinale* Roscoe*,* (SW 31)	Ginger (E), Yenjjeeluwa (W), Zinjibil (A)	Herb	Cultivated	Stem (tuber)	Bloat	Crushed plant stems or tubers are immersed in salted water after being crushed. The produced juice is then given orally once every day, up to recovery, in a glass or pan	Oral	Recovery	Bovine, ovine, caprine	81%

*Keys*; *A* Amharic, national language of the country, *E* English, *W* Wolaitic, regional language of the Wolaita people

### Collection habitat and growth pattern of medicinal plants.

**Fig. 3:**
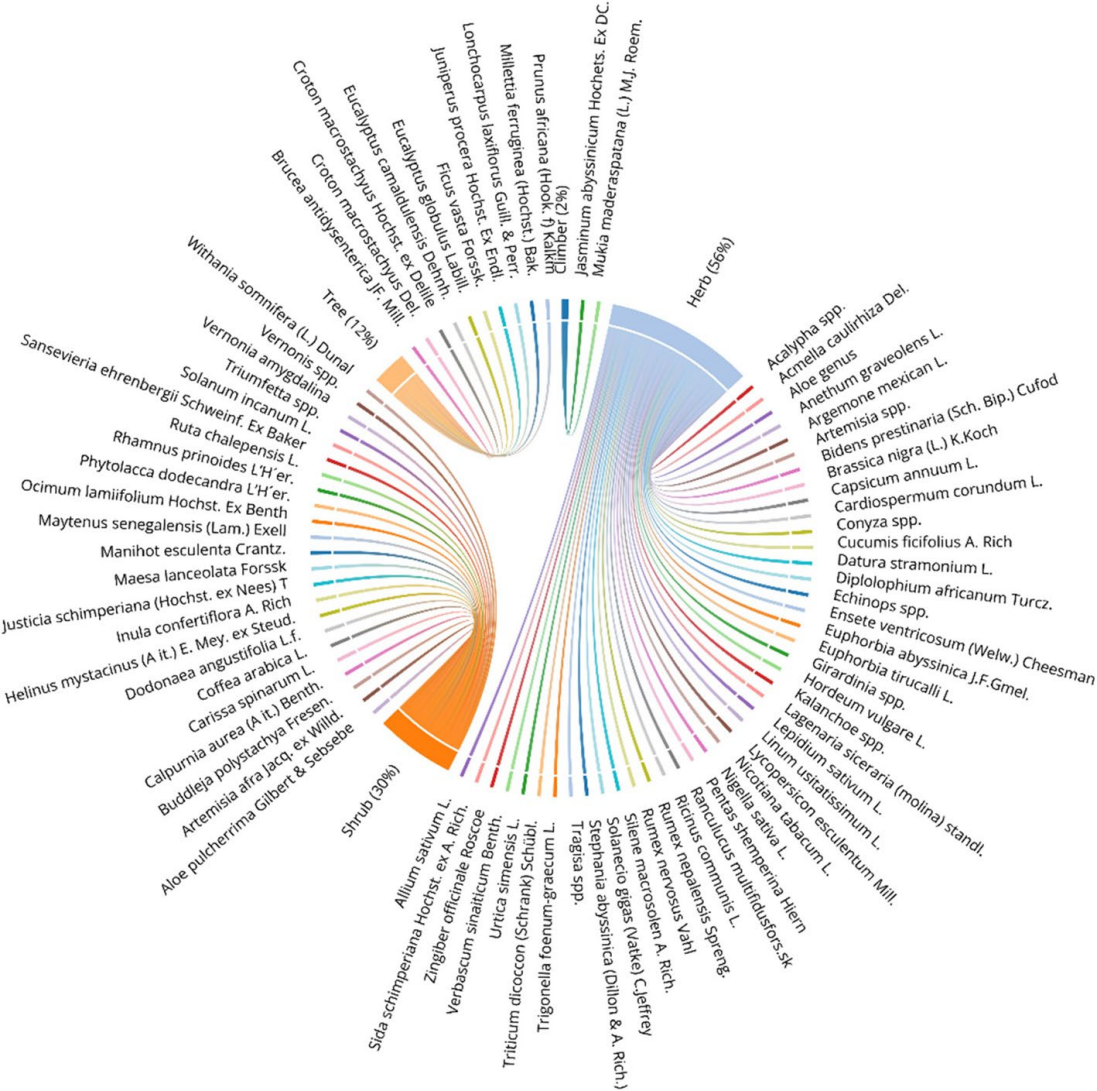
Medicinal Plants Growth habit

A total of 54 medicinal plants were identified, of which 62(66%) were collected from the wild, 24(26%) were grown in private gardens, and the remaining plant species 8(8%) was gathered from both agricultural and uncultivated wilderness areas. In terms of growth patterns, herbs made up 42(56%) of the higher plant species and were the most frequently gathered to treat livestock diseases, followed by shrubs 24(31%) and trees 10(12%) (Fig. 3).

### Use knowledge of medicinal plants among people

Despite the fact that male informants reported 133(65%) more medicinal plants than female respondents 72(35%), the difference was statistically insignificant (*p* > 0.05). Similar to this, there were reported medicinal plant differences between the community unable to read and write 64(80%) and educated groups 41(20%) that were statistically significant (*p* < 0.05). Compared to general informants, key informants knew a considerably (*p* < 0.05) a greater number of therapeutic plants (Table 2).

**Table 2:** Statistical test of knowledge among different groups of informants on average number of medicinal plants reported

Parameters used	Groups of informants	N	%	*P* value
Gender	Male	133	64.87	0.53
	Female	72	35.12	
Age	Youngest age range (20–39 years old)	37	10.04	0.000
	Senior group (40–85 years old)	168	81.95	
Educational level	Illiterate	164	80	0.01
	Educated	41	20	
Informant category	Key informants	130	63.41	0.003
	General informants	75	36.58	
Years of experience producing livestock	5–10	55	26.83	0.000
	11–20	65	31.7	
	21–30	70	34.14	
	Above 30	15	7.31	
Method of raising livestock	Subsistence	165	80.48	0.002
	Commercial	40	19.51	
The way that animals are treated	Medicinal plants only	48	23.41	0.01
	Combination of medicinal plants and conventional medicine	157	76.58	

*N* number of study participants; significance difference (*p* < 0.05)

### Acquiring and sharing the knowledge of native medicinal plants

From a total of 205 practitioners, 179 (87%) said they had heard stories about medicinal plants from members of their family, especially their father and grandparents, in a very private way. The remaining 15 (7%) and 11 (6%) picked up knowledge of medicinal plants from reading various sources, and trial-and-error, respectively.

### Distribution of medicinal plants

**Fig. 4:**
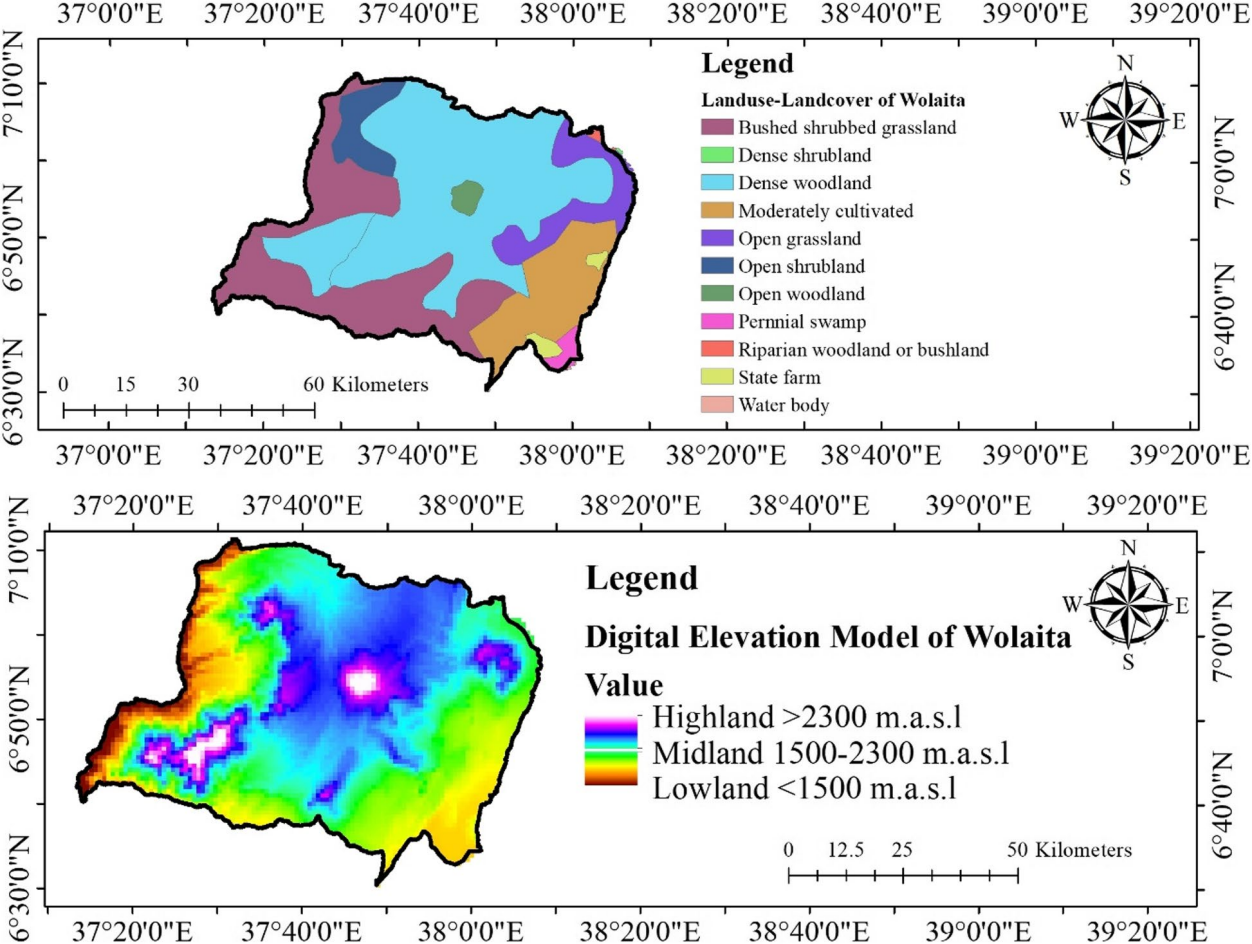
Land use – Land cover and digital elevation model (DEM) of Wolaita Zone (Source: AcrMap10.4.1 by Abenezer Wendimu)

**Fig. 5:**
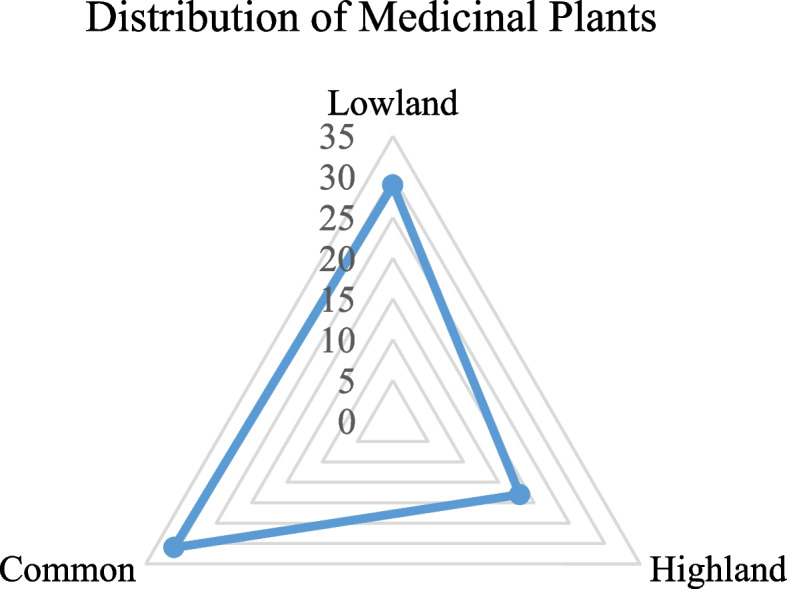
Distribution of medicinal plants in terms of altitude

The land use and land cover (LULC) data of the study area comprises more than 10 different flora distribution classes (Fig. 4, upper). The habitat of the medicinal plants in the whole Wolaita can be divided into three parts in terms altitude—lowlands (Lowland < 1500 m.a.s.l; near and around villages, collection time within a day, collection areas easily accessible) and highlands (Highland > 2300 m.a.s.l; far from villages, collection takes more than a day, collection areas remote and hard to access) and midland (Midland 1500–2300 m.a.s.l) small villages near the district towns, collection takes not more than a day, collection areas near and accessible) (Fig. 4, lower). Out of the reported species, 29 species are found in the lowlands and 18 species were found in the highlands with 31 species common in lowlands and highlands (Fig. 5). Generally, collection of highland medicinal plants is difficult and hence storage of such species for further use is common practice in the study area.

**Fig. 6:**
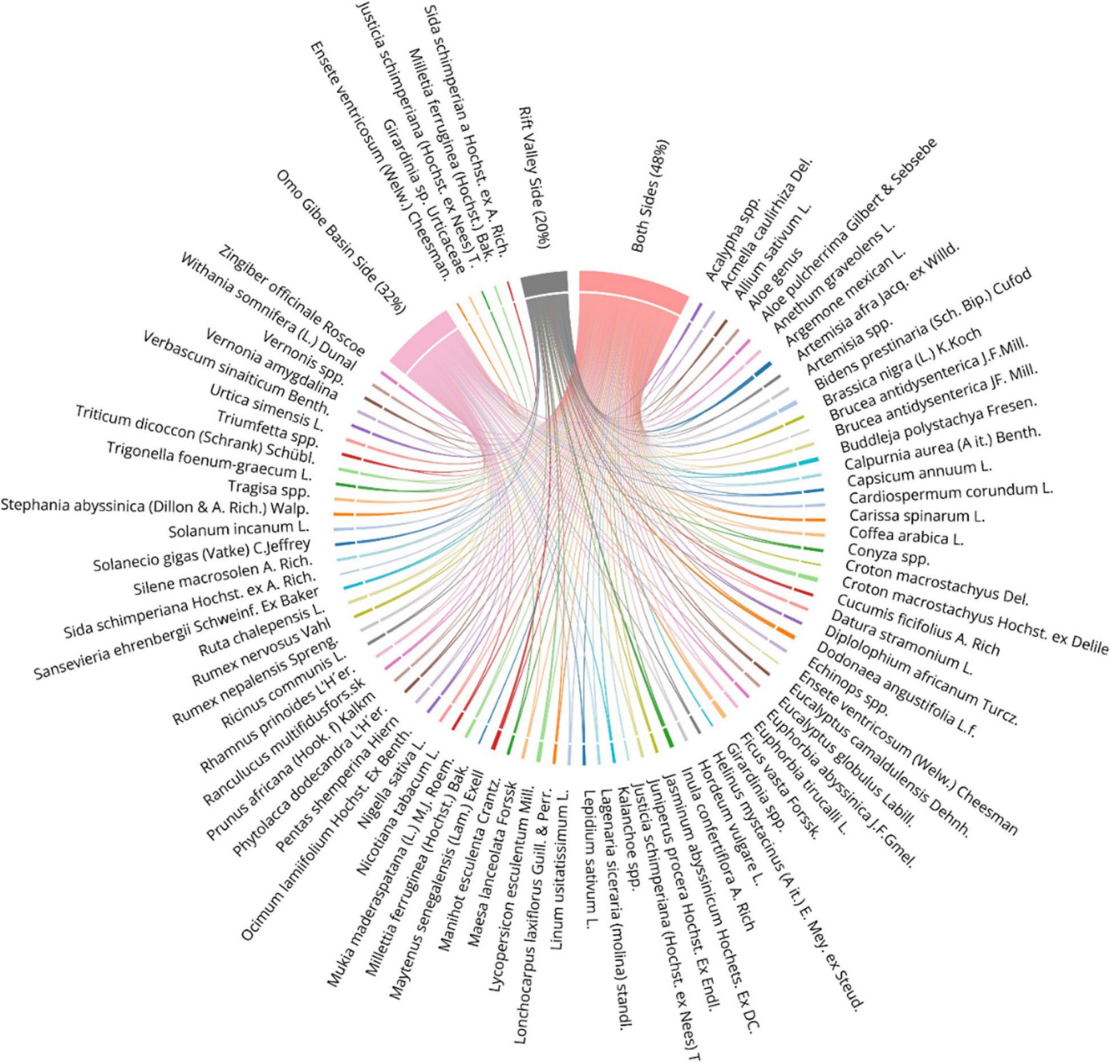
Distribution of medicinal plants in terms of drainage basins

The distribution of medicinal plants in terms of major drainage basins in Wolaita revealed that, Omo-Gibe basin holds about 32% of total collection of medicinal plants where as 20% were collected from Rift Valley basin. A significant amount of medicinal plants overlap were recorded from the two basins with 48% of collections common to both basins (Fig. 6).

### Aspects of animal disease in the study area

**Fig. 7:**
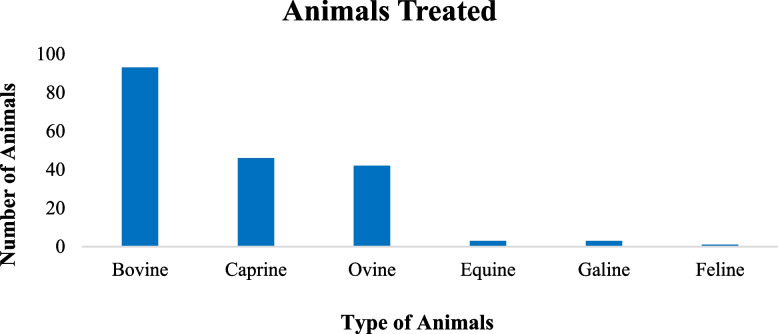
Animals treated by traditional medical preparations in the study area

This study found 55 different forms of animal illnesses. Highest number of species (16) were prescribed to treat blackleg followed by 10 species to bloat, 8 Trypanosomosis, and 7 each in colic and leech infestation (Table 3). Practitioners noted that they might utilize one or several medicinal plant species to treat a certain type of sickness. Endoparasite infestation, blackleg, and colic were the most common types of diseases treated by 9 medicinal plant species. In the Wolaita zone, the therapeutic indication of medicinal plant-based medicines included all livestock species. Medicinal plant cures were most frequently prescribed for Bovine ailments 93(49%), followed by Caprine 46(24%), Ovine 42(22%), Equine 3(2%), Galline 3(2%), and Feline 1(1%) aliments (Fig. 7).

**Table 3:**
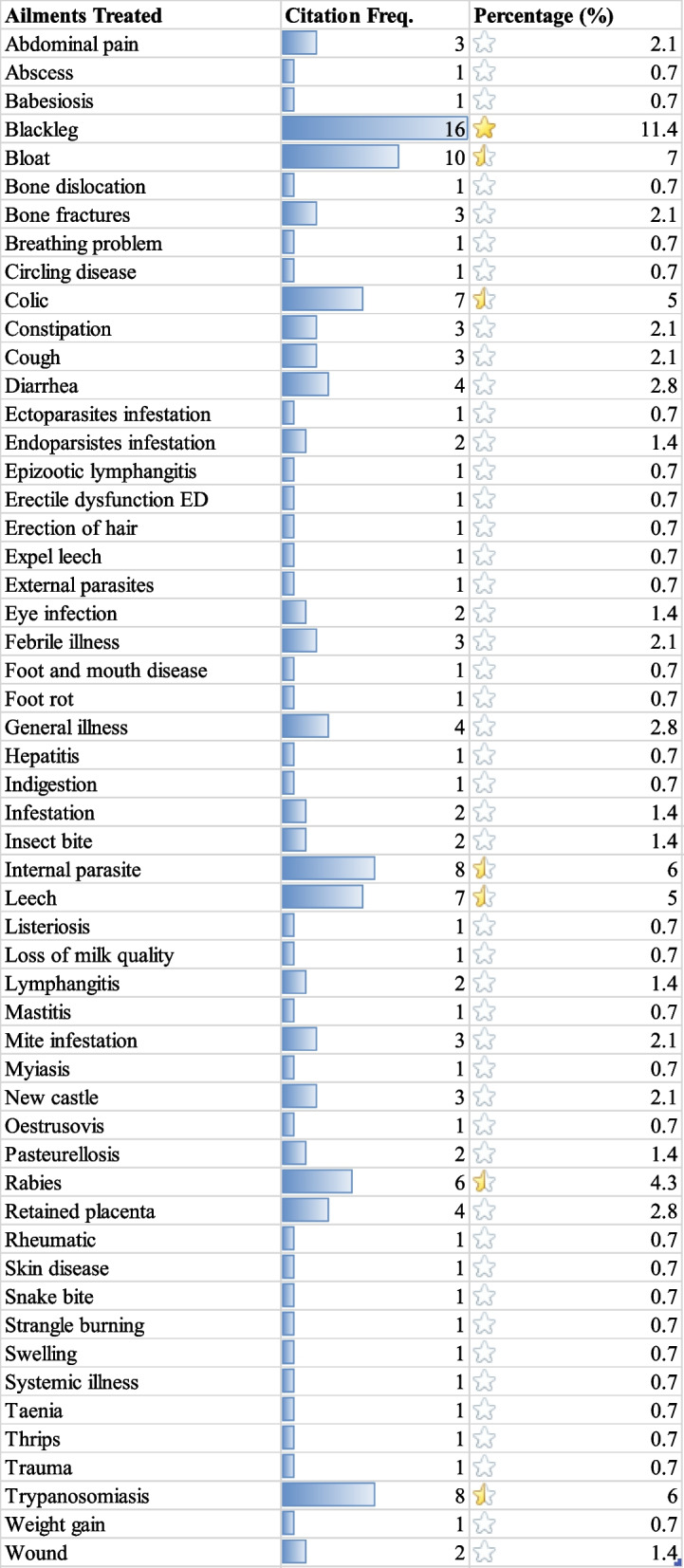
Ailments treated

### Parts of the medicinal plants utilized in the concoction of herbal remedies

**Fig. 8:**
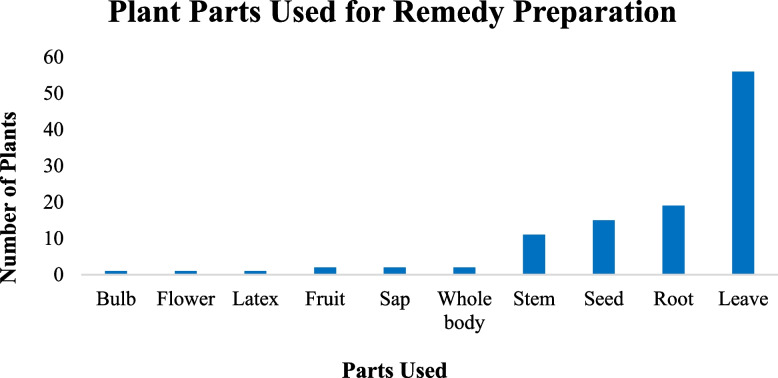
Plant parts used for preparation of the remedies

In the study area, leaves 55(52%) were the most often employed plant components in the preparation of remedies, followed by root 18(17%), seed 14(13%), and stems 10(9%). The remaining fruit, sap and whole parts of the plants contributed for 2% and 1% of the remedy preparation were prepared from bulb, flower and latex (Fig. 8). Regarding the state of the plant parts, freshly harvested plant parts dominated (80%), with the remaining 20% being used in both freshly and in a dried form.

### The routes of remedy administration

**Fig. 9:**
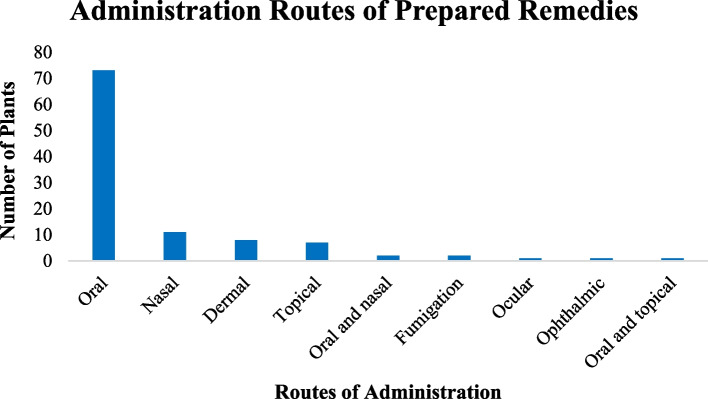
The administration routes of ethnobotanical preparations

The target animal and the type of sickness determined how to administer ethnobotanical preparations. In the study area, the most common modes of administration were nasal, oral, topically/dermally, and intraocularly, and others include applying the medication directly to a fresh lesion or cut. Oral 73(69%) and nasal 11(10%) routes were the most frequently used delivery methods followed by dermal 8(7%) and topical 7(7%) administration routes. Ethnomedicines applied via the ophthalmic and ocular modes of administration routes were the least cited routes (Fig. 9).

### Informant consensus factor

**Fig. 10:**
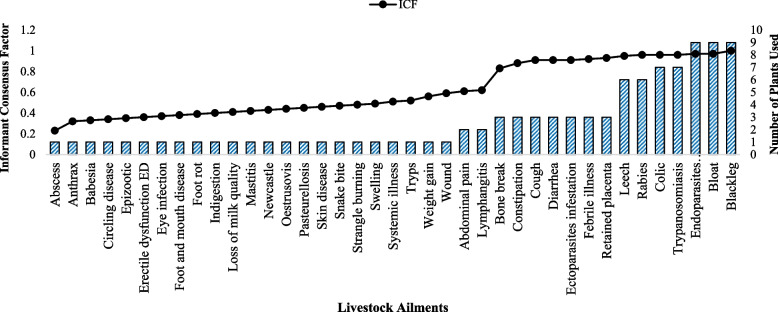
Livestock ailments in the study district along with the Consensus Factor of Informants (ICF)

Blackleg, bloat, and endoparsistes each had the highest values of the consensus factor among the informants, which were followed by trypanosomiasis (0.8) and colic (0.79) (Fig. 10). Leech and rabies took the next two spots (0.75 each).

### Fidelity level

*Hordeum vulgare* L. had the highest fidelity level (FL = 98%) for treating bone fractures followed by *Acalypha* spp. for treating bloat (FL = 92%) (Table 2).

### Preference ranking

According to data collected from six key informants, *Withania somnifera* was the most potent remedy for treating blackleg. *Tragisa* spp. and *Kalanchoe* spp. were the next most efficient therapeutic plants. *Ocimum lamiifolium* and *Prunus africana* were, in comparison, the least effective plants for medicinal purposes, according to the data gathered from six key informants (Table 4).

**Table 4:** Preference ranking medicinal plants to treat blackleg

No	Medicinal plants name	Informants							
		I1	I2	I3	I4	I5	I6	Total score	Ranking
1	*Ocimum lamiifolium*	0	2	2	1	4	2	11	9th
2	*Prunus africana*	0	2	2	3	4	3	14	8th
3	*Sida schimperian* a	2	2	5	2	2	3	16	7th
4	*Stephania abyssinica*	4	5	3	4	1	3	20	6th
5	*Croton macrostachyus*	3	5	4	4	2	3	21	5th
6	*Allium sativum L*	3	5	4	4	3	3	22	4th
7	Kalanchoe spp	3	5	4	4	5	3	24	3rd
8	Tragisa spp.	3	5	4	4	5	3	24	2nd
9	*Withania somnifera*	4	4	5	5	5	2	25	1st

### Medicinal plant threats in the area

**Fig. 11:**
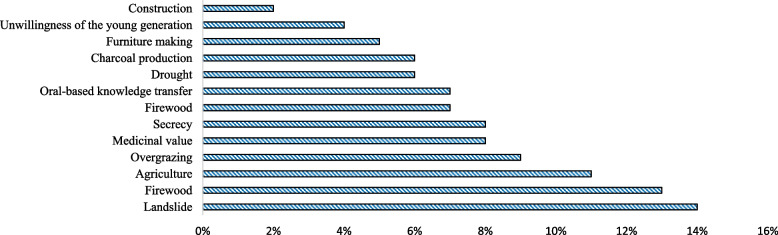
Medicinal plants conservation threats in the study area

Both natural factors (such as drought and landslides) and anthropogenic factors (firewood, overgrazing, agricultural expansion, construction, and medicinal use) have an impact on the survival of medicinal plants in the study district. The main dangers to medicinal plants in the study district were the expansion of agriculture, followed by drought and development (Fig. 11).

## Discussion

Livestock is essential to the livelihoods of the inhabitants of the study districts for a variety of reasons, including crop production, draft power, marketing, and revenue generating. Despite the fact that knowledge differed depending on the age and sex group, this causes the residents of the district to have the knowledge to defend their animals from a variety of illnesses using therapeutic plants. There were just 64.87% of women respondents in this survey, whereas there were 35.12% men. The elders may pass on their knowledge to their older son or to their preferred son rather than their daughter, which could be the cause of this. A different Ethiopian region has also observed similar results [37, 38]. The reason behind this preference for passing on knowledge to a male heir rather than a female one may be rooted in traditional gender roles and societal norms. In many cultures, especially patriarchal ones, sons are often seen as the ones who will carry on the family legacy and continue the family line, while daughters are expected to marry into another family. Additionally, there may be a belief that certain types of knowledge are better suited for men to carry on, such as medicinal or magical practices that require physical strength or are traditionally performed by men. It is important to note that these beliefs are based on outdated gender stereotypes and should not be used to justify discrimination against female family members. Even though, on average, males used slightly more medicinal herbs than women, the differences were not that much big and statistically insignificant (*p* = 0.53). Other factors may be influencing the higher usage of medicinal herbs among males, but further research is needed to determine these factor.. This finding was consistent with the conclusions reached by Yigezu et al. [39]. The study also revealed that older groups of informants reported a considerably larger average number of therapeutic plants than the youngest group (*p* < 0.05). The main factors to this notable disparity are the growth of modern medicine and the younger age groups' disinterest in traditional medications. There could be other factors at play, such as differences in cultural upbringing, access to traditional medicine, or differing beliefs about the effectiveness of traditional remedies.

In addition, the youthful group lacks interest due to the seasonal scarcity of therapeutic plants and their hard harvesting. As a result, a decline in positive attitudes toward traditional medicine is a sign that knowledge of these practices and the species of medicinal plants is eroding. The research by Yigezu et al. [39] and Lulekal et al. [40] got the same results. In accordance, significantly (*p* < 0.05) more medicinal plants were reported by illiterate respondents than by educated respondents. This is because respondents who were educated preferred modern treatment and paid less attention to traditional medicine. In turn, this leads to a decline in medical expertise in the following generation. By Birhan et al. [8], the same conclusion was reported. Key informants could report significantly more medicinal plants than general informants (*p* < 0.05) as a result of their experience.

The identification and documentation of 78 ethnoveterinary medicinal plant species, including their scientific and local names, habits, methods of preparation, and used components, was included in the present study. The dominant families were Euphorbiaceae and Asteraceae. Ethiopia's Amhara region, according to Lulekal et al. [40], likewise exhibited a predominance of the Asteraceae family. However, the family Solanaceae has been found in various regions of Ethiopia [11, 18, 41, 42], which goes against this study. The district is home to a diverse population, according to this result. Furthermore, the preference for native and endemic plants with therapeutic properties shows that people don't have contemporary knowledge but rather has a lengthy history and is handed down from one generation to the next over an extended period of time. This finding was consistent with studies by Mengesha [43] and Lulekal et al. [40].

The bulk of plant growth forms investigated for medicinal purposes were herbs; shrubs came in second, and trees and climbers next in sequential order of species. In a different region of Ethiopia, a high consumption of shrubs for their therapeutic benefits was noted [18, 41, 44]. This can occur as a result of the relatively abundant herb availability for practitioners in the study districts. According to other findings [37, 45, 46], shrubs predominate.

The majority of the plants in this study—32 (61.5%)—were gathered from the wild, while 11 (25%) were taken from backyard gardens, and the remaining 7 (13.5%) species were found in both backyard and wild habitats. The results of other authors [18, 40, 44, 46] were consistent with this finding. This indicates that growing plants in a home garden for therapeutic reasons is quite uncommon in the research area. This decreased practice of growing medicinal plants in backyard gardens results in a lack of those plants throughout the year as desired by practitioners.

Many medicinally important plant species were reported to be found in the mid-altitudinal ranges (1500–2300 m.a.s.l) for the ollection areas were near and accessible. This is in line with Kunwar and Bussmann [47] who reported an increase of medicinal plant species with increasing altitude up to about 2000 m.a.s.l. The medicinal plant diversity corresponds with total richness of plant diversity [48], however, the high value species were reported from the highland areas.

Although different plant parts have been used to treat various illnesses, leaves and seeds were the two plant parts most frequently used in the study district. This result is consistent with research conducted by Tekle [49] in the southern Ethiopian Amaro special district, the western Ethiopia Horro Gudurru district [18], and the eastern Harerghe Melkabello district [41]. It disagrees, however, with studies done by Jima and Megersa [50] in the Berbere District of Oromia region and by Seid [44] in the Amhara region's Enarj Enawega District's east Gojjam Zone [8]. The persistence of plants in their native environment is not significantly impacted using leaves for medication. However, the use of roots for medicinal purposes could result in the extinction of certain species from their natural habitats as well as the native plant medicine expertise is being lost. The majority of practitioners in the research district like plants in fresh 60 (80%) circumstances. The results of Chekole et al. [51] are consistent with this finding. The harvesting of fresh plant material used by practitioners to make medicines during the dry season may cause the plants' species to be stripped.

Oral form of administration accounted for 62 (74%) of all administrations, followed by nasal mode of administration (13%) and cutaneous mode of administration (8%). This result was consistent with that of Yigezu et al. [39]. There is no standard unit of measuring for plant remedies in the study district; instead, people use their own system of measurement. The medication dosage is based on the age, breed, and size of the animals that are receiving treatment. Other authors reported the same results [42, 52–56].

Blackleg (0.82), general sickness (0.8), and pasteurellosis (0.79), according to informants' consensus factors (ICF), had the highest values. Blackleg received the highest plant citation at the time, 10(30.3%), followed by general disease 7(21.21%). This makes it very evident that blackleg is a widespread and well-known illness in the studied area. There are 10 ethnoveterinary medicinal herbs that can be used to treat this condition. *Vernonia amygdalina* was the most popular medicinal plant species for treating blackleg, followed by *Cucumis ficifolius* and *Solanecio gigas*. According to Mengesha and Dessie [53] and Tadesse et al. [54], different districts used the same ethnoveterinary medicinal herbs that were identified in the research districts to treat blackleg.

In general, the research area has a high biodiversity, and the locals have a wealth of conventional knowledge concerning the use of herbal remedies to treat certain livestock diseases. A variety of indigenous plant species can be found in the study region. The findings of this investigation demonstrated that the district's expertise and therapeutic plants are vulnerable. Therefore, it necessitates special consideration from the public, the government, and all stakeholders.

The cultural interpretation of medicinal plants and diseases in the Wolaita region offers a fascinating glimpse into the rich heritage and traditional knowledge of the local community. For generations, the people of Wolaita have developed a profound understanding of the medicinal properties of various plants, which they have used to treat and manage a wide range of diseases and ailments. The traditional healers, known as "hiillaa," play a significant role in preserving this knowledge and administering remedies derived from the region's diverse flora. These healers possess an intimate understanding of both the physical and spiritual aspects of healing, often incorporating rituals, prayers, and incantations into their practices. In the study area, specific diseases are attributed to spiritual or supernatural causes, leading to the use of specific plants and rituals aimed at appeasing or combatting these metaphysical influences. The cultural interpretation of medicinal plants and diseases in Wolaita intertwines the deep-rooted beliefs, values, and practices of the local population, showcasing their respect for nature and their reliance on traditional wisdom passed down through generations.

### Limitation of the study

The study was conducted in small portions of the Omo-Gibe and Rift Valley basins in Ethiopia, which may not fully represent the entire ethnobotanical use of plants in treating livestock ailments of these basins. Future studies should aim to include a more comprehensive assessment of the ethnobotanical use of plants in these basins to provide a more holistic understanding of medicinal plant species diversity, use, distribution, and abundance in the area.

## Conclusions

The communities that were chosen are mainly rural in character, and livestock farmers are looking into the local biodiversity and indigenous knowledge systems to satisfy the demands of animal health and productivity. In the study region, 78 ethnoveterinary species of medicinal plants from 28 distinct families were discovered. The Euphorbiaceae and Asteraceae plant families made up the majority of those that were noted. Herbs were the most frequently collected to cure livestock sickness and made up higher plant species in terms of growth patterns. Most of the plants were collected from the wild. According to practitioners, they would use one or several therapeutic plant species depending on the disease. Nine different species of medicinal plants were used to cure the most frequent ailments, including colic, blackleg, and endoparasite infestation. Bovine ailments were most frequently treated using medicinal plant remedies. The plant's fresh leaves were the plant parts that were most frequently used to make medicines. The most popular remedy administrative methods in the study area included the nose, mouth, skin, eyes, and others, including applying the drug directly to a fresh lesion or cut. Blackleg, bloat, and endoparsistes had the highest values of the consensus factor among the informants, according to the data. According to data collected from six important informants, *Withania somnifera* was the most potent remedy for treating blackleg. The primary threats to botanical medicine in the study region were the expansion of agriculture, followed by drought and development. Despite the fact that the research region in the districts of the Wolaita Zone was proven to be rich in a variety of medicinal plants, there are currently few attempts being made to look at the native knowledge and plants associated with them. To prevent such losses, communities at large along with accountable organizations must protect therapeutic plants. It's also important to choose plants for further study that have a high potential based on the relevant ethnobotanical indices, such as phytochemical analysis and pharmacological and toxicological investigations. In general, the research area has a high biodiversity, and the locals have a wealth of conventional knowledge concerning the use of herbal remedies to treat certain livestock diseases. A variety of indigenous plant species can be found in the study region. The findings of this investigation demonstrated that the district's expertise and therapeutic plants are vulnerable. Therefore, it necessitates special consideration from the public, the government, and all stakeholders.

### Supplementary Information

**Supplementary Material 1.**

## Data Availability

The data used to support the findings of this study are included within the supplementary information file(s).
