# Supplementary material for: Medicinal ethnoveterinary plants used for treating livestock ailments in the omo-gibe and rift valley basins of Ethiopia
Source: BMC Vet Res. 2024 Apr 30;20:166. doi: 10.1186/s12917-024-04019-6 (PMC11059770; doi:10.1186/s12917-024-04019-6)
Supplement: Supplementary file 1 — Supplementary Material 1. [file 12917_2024_4019_MOESM1_ESM.docx]

**S1 Appendix. English version of questioner.**

**Questionnaire**: Semi-structured questionnaire (English Version)

The aim of this questioner is to identify the types of animal ailments which occur in the study district, and to identify the effective medicinal plants to treat these ailments. In addition to these, it also important to check the status of conservation habit of the people in the district. Finally, it is important to document medicinal plant knowledge of the people and to plan continuity of this golden knowledge. So please give appropriate answer for these questioners. Thanks in advance for your response.

Village:____________

Date: ___________

Informant No.:___________

**Participants’Information:**

Name: ___________________________________ Age: _____ Gender: Male_____ Female____

Education status: ___________ Marital Status: Single____ Married_____ No. of children: ____

Years of residence: ________Occupation: ____________________Income:_____________

Religion: __________Others:______________________________________________

**Research questions:**

1. What are the major medicinal plants used to treat different aliments?
2. Which part of the plant is used as remedy? (Leaf/Stem/Root/Flower/Bark/Fruit/Seed).
3. What are the methods of preparation of the medicinal plants? (Crushed/ pounded/ Powdered/ Concoction/ Decoction/ Infusion/ mixed with others).
4. Forms of medicinal plants used (Fresh/Dryform).
5. How is the dosage and way of administration of medicinal plants? (Oral/Nasal/Dermal)
6. Time of medicinal plants collection (Morning/Afternoon/Night).
7. The other uses of medicinal plants (Food/Fodder/Fence/Firewood).
8. Storage and conservation of medicinal plants.
9. Source and transfer of indigenous knowledge (Father/Mother/Friends).
10. Animal species that received treatment (Bovine/Ovine/Caprine/Equine/Galine)
11. The care and supervision given to animals

____________________________________________________________

1. How the services of ethnopractitioners are compensated

_________________________________________________________________

1. The methods by which practitioners of ethnoveterinary medicine exchange knowledge

____________________________________________________________

1. Cattle illnesses and/or disorders treated

____________________________________________________________

1. Utilized plants and/or plant products, along with their form or state

____________________________________________________________

1. State of the plant or any products made from plants that are utilized for treatment

____________________________________________________________

1. Variables that affect the status of the plant and/or the plant products employed in treatment

____________________________________________________________

1. Actions being done about the condition of the plant and/or plant products used as medicine

_________________________________________________________________

1. The difficulties facing the field of ethnoveterinary medicine

_________________________________________________________________

1. The interviewee's own views on the practice of ethnoveterinary medicine

_________________________________________________________________

1. What actions should be taken to enhance local traditional veterinary services

_____________________________________________________________
